# Detection and identification of pathogens using agents targeting the bacterial cell wall

**DOI:** 10.1007/s12223-025-01379-w

**Published:** 2025-12-11

**Authors:** Aliaksandr Zhydzetski, Zuzanna Głowacka-Grzyb, Kinga Chlebicka, Benedykt Władyka

**Affiliations:** 1 Department of Analytical Biochemistry, Faculty of Biochemistry, Biophysics and Biotechnology, Jagiellonian University, Gronostajowa St. 7, Cracow, 30-348 Poland; 2 Doctoral School of Exact and Natural Sciences, Jagiellonian University, Prof. St. Łojasiewicza St. 11, Cracow, 30-348 Poland

**Keywords:** Bacterial cell detection and identification, Bacterial cell wall structure, Cell wall-binding domain (CBD), CBD-conjugates, Receptor binding proteins (RBPs)

## Abstract

The widespread emergence of multidrug-resistant pathogenic bacteria across various environments, healthcare settings, and food industries, combined with the development of new methods to combat them, highlights the need for more precise, rapid, and cost-effective pathogen detection techniques. This is especially important for clinically relevant pathogens, as it allows treatment to begin as quickly as possible, enables more effective targeted therapies to be chosen, helps preserve the effectiveness of current antibacterial agents, and prevents infections from water- and foodborne bacterial pathogens. Currently, many methods can accurately identify bacteria at the species or strain level and determine their antibiotic resistance. However, most of these techniques require sample preparation and cell culture beforehand, which can be time-consuming and labor-intensive. This review aims to highlight approaches that focus on identifying bacterial cells—especially pathogenic groups—based on their surface properties. This includes agents such as antibodies, whole phage particles, phage receptor binding proteins, cell wall-binding domains of peptidoglycan hydrolases, and functionalized magnetic nanoparticles. These agents can bind to and recognize peptidoglycan, parts of it, and other cell wall components. Developing detection kits based on these agents could enable the rapid detection of pathogenic bacteria from genera such as *Acinetobacter*, *Bacillus*, *Campylobacter*, *Clostridium*, *Enterococcus*, *Klebsiella*, *Listeria*, *Pseudomonas*, *Salmonella*, *Shigella*, *Staphylococcus*, *Streptococcus*, *Vibrio*, and *Yersinia*. These methods also offer the potential to distinguish these infectious pathogens from each other and from bacteria of the natural microbiota. Detection typically takes from a few minutes to several hours, with a broad detection range depending on the pathogen species, the detecting agent, and the technique used.

## Introduction

The spread and significant impact of bacterial infections, especially in the context of multidrug resistance, on human health and the economy demand fast and reliable methods for detecting and identifying pathogenic bacteria to enable quick treatment. This is essential not only for healthcare but also for food and water safety, animal and plant health, environmental monitoring, and clinical diagnosis, all of which are critical for safeguarding public health (Rajapaksha et al. 2019; Canciu et al. 2021; Deusenbery et al. 2021). Currently, many bacterial detection techniques fall into two categories: direct culture-independent detection and traditional indirect culture-based methods (Wang & Salazar 2016).

Culture-based methods for detecting bacterial pathogens were among the earliest developed techniques, including isolating and culturing microorganisms on agar plates, followed by analyzing their various phenotypic characteristics such as Gram staining. Phenotypic methods for genus and species identification depend on a characteristic protein fingerprint derived from unique biochemical pathways and processes, which can be detected through biochemical analytical techniques, microscopy methods, and chromogenic media (Xu et al. 2016; Braga et al. 2013; Ferone et al. 2020). Detection with chromogenic media relies on the development of color or fluorescence after hydrolysis of colorless chromogenic or fluorogenic substrates by specific enzymes expressed by the test bacteria (Galat et al. 2016; Bajoria et al. 2019). Different microscopy techniques, especially when combined with machine learning, are essential tools for bacterial identification. They enable the assessment of morphological and structural properties such as size, shape, and motility (He et al. 2018b; Muller et al. 2018). Biochemical techniques for phenotypic testing include pH-based reactions, enzyme profile assays, carbon source utilization, and carbohydrate metabolism (acid production and end product analysis). They also include tests for protein, amino acid, lipid metabolism, and other metabolites, as well as phenotypic tests for cell wall receptors, lysozyme susceptibility, and bile solubility (Franco-Duarte et al. 2019). These biochemical assays can be performed manually or with automated systems. Like chromogenic media and microscopy, which are not entirely specific, they can be combined with culture-independent molecular methods for further, more accurate confirmation of species identity.

Although inexpensive and easy to perform, the main drawbacks of the aforementioned culture-based methods are that they are labor-intensive and time-consuming, involving media preparation, dilution, plating, incubation, counting, isolation, and characterization. Usually, it takes 2 − 3 days, or even several weeks in the case of *Mycobacterium tuberculosis*, to go from initial identification to confirming the pathogen’s species (Zhao et al. 2014; Varadi et al. 2017). Additionally, these methods can yield misleading identification and false positive results, and they cannot detect several important but non-culturable bacterial pathogen cells (Franco-Duarte et al. 2019).

Another group of high-sensitivity and specificity methods used for detecting, identifying, characterizing, and typing bacteria in clinical and research settings is the culture-independent, molecular-based techniques. These methods rely on analyzing genomic markers associated with nucleic acid sequences (particularly the conserved rRNA genes) or other cellular molecules (Wang et al. 2016; Varadi et al. 2017). Nucleic acid-based methods detect specific DNA or RNA sequences in target pathogens and include techniques such as hybridization, amplification, and sequencing. Hybridization-based detection includes fluorescence in situ hybridization (FISH) and peptide nucleic acid fluorescence in situ hybridization (PNA-FISH). Amplification methods include simple polymerase chain reaction (PCR), often combined with product sequencing, quantitative real-time PCR (qPCR), reverse transcriptase real-time PCR (RT-qPCR), nucleic acid sequence-based amplification (NASBA), loop-mediated isothermal amplification (LAMP), high-resolution melting (HRM), random amplification of polymorphic DNA (RAPD-PCR), restriction fragment length polymorphism (RFLP), and amplified fragment length polymorphism (AFLP). Nucleic acid-based techniques also include PCR-Ribotyping, DNA microarrays (gene chip technology), and whole genome sequencing (WGS) (Varadi et al. 2017; Buszewski et al. 2017).

Multiomics approaches, such as metagenomics, proteomics, and metabolomics, often use matrix-assisted laser desorption/ionization time-of-flight (MALDI-TOF) or electrospray ionization (ESI) mass spectrometry (MS) combined with gas chromatography (GC), high-performance liquid chromatography (HPLC), and other techniques. These methods compare fingerprint MS spectra of characteristic proteins (mostly ribosomal proteins) from sample bacteria with patterns in online MS databases. They are now more commonly used to identify bacterial genera and species. Besides proteins, spectral patterns of biomolecules like lipids, carbohydrates, and amino acids can also be used for bacterial identification. For a more thorough review of molecular diagnostic techniques, we refer the reader to the excellent reviews by Varadi et al. (2017), Franco-Duarte et al. (2019) and Law et al. (2015).

However, despite the benefits of molecular methods, such as being reproducible, automated, highly specific, sensitive, relatively quick, labor-saving, and capable of detecting non-culturable organisms, these techniques also have several limitations and still need further improvements. They often require pre-enrichment and pre-treatment steps, as well as expensive and specialized equipment, and skilled personnel. Additionally, they sometimes cannot distinguish between taxonomically related bacteria, are difficult to differentiate between viable and non-viable cells, and pose challenges in RNA handling. These methods can be impacted by PCR inhibitors and interference from other compounds because most clinical, food, or environmental samples are complex matrices, which can lead to cross-reactivity and/or false-negative results (Law et al. 2015).

Therefore, rapid and reliable detection of bacterial species, followed by more precise identification of specific serotypes using molecular methods, is crucial for early diagnosis and effective treatment. For this purpose, the bacterial cell wall—being the first line of defense—can serve as the primary target not only for attacking and destroying bacteria with subsequent cell death but also for bacterial detection and identification (Zhydzetski et al. 2024). In this review, we consider the bacterial cell wall and its components as fingerprints and primary targets for determining bacterial genus and species specificity, along with molecules and systems that could be employed for this purpose. Naturally, these methods and biorecognition molecules should meet criteria such as being fast, enabling discrimination of the target pathogen from background microbiota, possessing high physicochemical, and enzymatic resistance, providing high specificity, sensitivity, and selectivity, having a low probability of reaction inhibition due to sample impurities, being non-cross-reactive and straightforward, and being inexpensive and easy to produce because many areas in need of such diagnostic systems cannot afford expensive tests (Costa et al. 2023).

## Bacterial cell wall structure

**Fig. 1:**
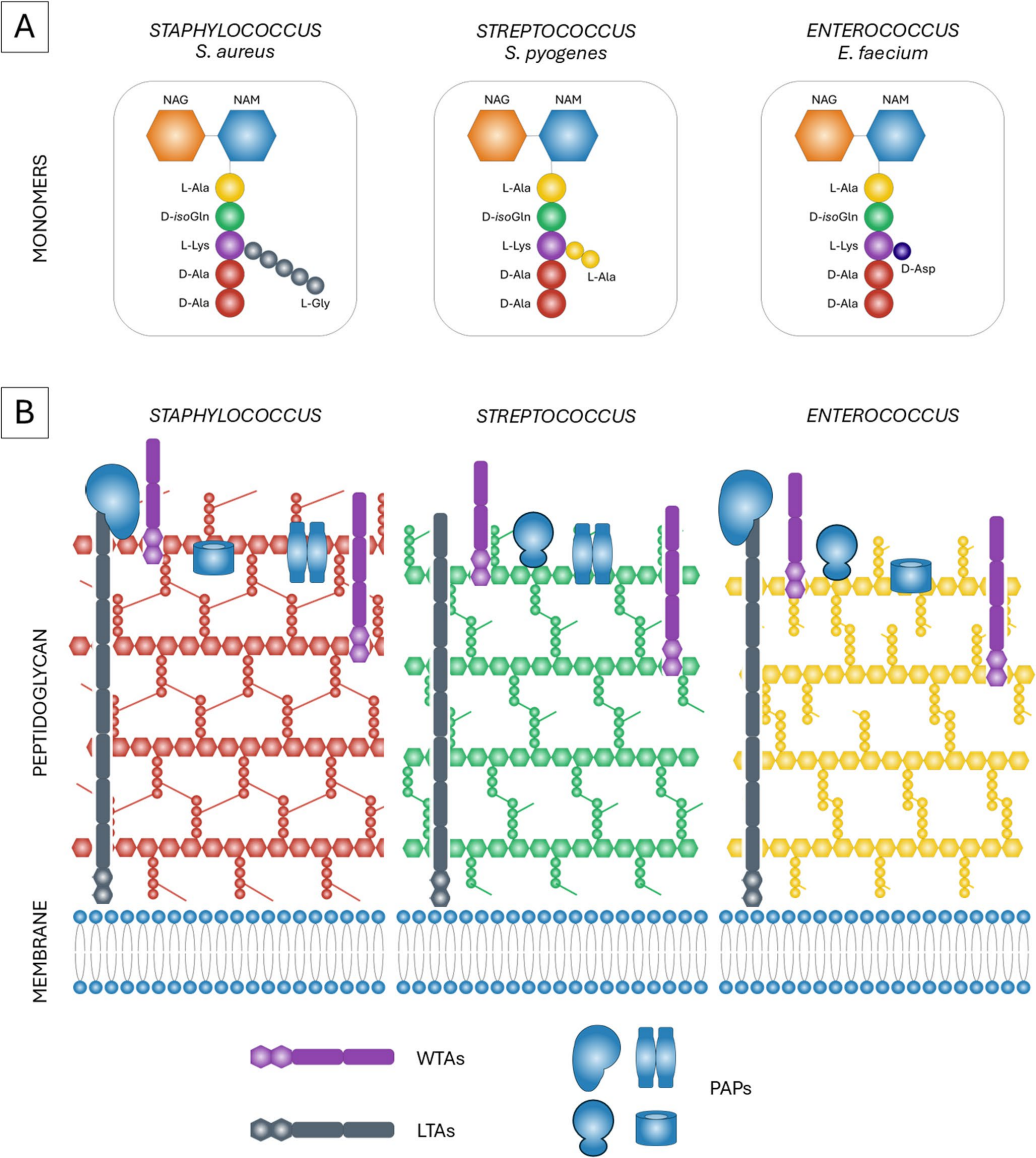
Schematic representation of Gram-positive peptidoglycan varietes. **A.** The peptidoglycans of *Staphylococcus*, *Streptococcus* and *Enterococcus* consist of similar monomers, containing NAG and NAM backbone and stem peptide with adjacent interpeptide bridge. In *S.aureus* a pentaglycine bridge is observed, in *S. pyogenes* two L-Ala residues, and *E. faecium* contains D-Asp residue. **B.** The peptidoglycan is synthesized and crosslinked outside the cellular membrane forming linear strands connected together by interpeptide bridges. The staphylococcal peptidoglycan is easily distinguished by pentaglycine bridges. In *Streptococcus*, stem peptides are truncated to tri- and tetrapeptides by transpeptidases, and later crosslinked via dialanine bridges. The crosslinking rate of enterococcal peptodglycan is about 50%. All peptidoglycans contain teichoic acids anchored in either cellular membrane (LTAs) or to NAG residues in glycan strands (WTAs). A plethora of peptidoglycan associated proteins (PAPs) are bound to teichoic acids and glycan components, and their composition is often strain specific (i.e. allowing for serotyping in *Streptococcus*)

Based on the presence of an outer membrane and the thickness of the peptidoglycan (PG) layer, bacteria can be classified into Gram-negative and Gram-positive cells. Gram-negative bacteria have both a cytoplasmic and an outer membrane, which contains lipopolysaccharides (LPS). Gram-positive bacteria lack an outer membrane and therefore do not have LPS. However, the cell wall of Gram-positive cells contains polysaccharides such as wall teichoic acids (WTA), covalently attached to the peptidoglycan, and lipoteichoic acids (LTA), anchored into the cytoplasmic membrane (Fig. 1B). Peptidoglycan, a cross-linked polymeric meshwork found in almost all bacterial cells, is thinner in Gram-negative bacteria and thicker in Gram-positive bacteria. Despite this difference, Gram-negative and Gram-positive bacteria share a similar structure consisting of polysaccharide chains cross-linked with peptides (Auer and Weibel 2017; Campanero-Rhodes et al. 2020).

The peptidoglycan of Gram-positive bacteria is described based on the cell wall of the most prominent representative of the *Staphylococcus* genus – *Staphylococcus aureus*. It forms a multilamellar cross-linked structure with peptide chains attached to a glycan strand (Sutton et al. 2021). The disaccharide backbone consists of alternating residues of N-acetylmuramic acid (NAM) and N-acetylglucosamine (NAG). The D-lactoyl group of NAM is substituted by the first amino acid of an oligopeptide called the stem peptide (Vollmer et al. 2008). The stem peptide composition is unique, alternating between two to five amino acids. The standard sequence usually includes L-Ala, D-isoGln, L-Lys, and two D-Ala residues (Apostolos et al. 2022). In *S. aureus*, a pentaglycine bridge attaches to the ε-amino group of L-Lys, which then connects to the terminal D-Ala of another PG strand during cross-linking (Fig. 1A). Crosslinked PG creates a rigid, strong net that protects the bacterium from the environment (Irazoki et al. 2019). The PG composition and structure depend on nutrient availability. In a glycine-depleted environment, *S. aureus* does not crosslink due to the lack of a pentaglycine bridge but compensates for defective peptidoglycan by increasing PG thickness (Zhou and Cegelski 2012). PG can be further modified by O-acetylation of NAM at the C-6 hydroxyl group during the assembly of saccharides in the cytoplasm (Sychantha et al. 2018), enabling pathogenic bacteria to evade host innate immunity responses, especially lysozyme (Chang et al. 2017). The PG of *S. aureus* contains a major class of surface polymers called teichoic acids (TA). Depending on their localization, they are classified as wall teichoic acids (WTA) attached directly to PG, or lipoteichoic acid (LTA), anchored in the cytoplasmic membrane (Brignoli et al. 2022). Despite this distinction, WTA and LTA are involved in similar processes such as scaffolding for cell shape determination, cell division, cation binding, and are required for β-lactam resistance in MRSA (Swoboda et al. 2010; Brown et al. 2013). The staphylococcal PG is rich in various peptidoglycan-associated proteins anchored by sortase A (Chen et al. 2023). Over 20 types of these proteins are classified into five groups. Molecular surface components recognizing adhesive matrix molecules (MSCRAMMs) facilitate bacterial cell binding and clumping (Foster 2019a). Near iron transporter (NEAT) motif proteins acquire heme and iron (Honsa et al. 2014). G5-E repeat family and legume lectin-containing family promote cell aggregation (Foster 2019b). Three helical bundle motifs, including protein A, help modulate its conformation to bind various ligands (Deis et al. 2015). The last group includes LcpA (Li et al. 2020) and TarM (Koc et al. 2015), which attach teichoic acids to PG, and DivIB, which is essential for cell growth and division (Bottomley et al. 2014).

Similarly to PG of *S. aureus*, the streptococcal cell wall also contains a glycan scaffold of NAM and NAG, some of which are modified by O-acetylation and N-deacetylation. The most abundant stem peptide found in *S. pneumoniae* is an Ala-isoGln-Lys tripeptide (Vollmer et al. 2019; Greene et al. 2015), although both linear and branched stem peptides are present. The murMN operon encodes proteins involved in the biosynthesis of branched stem peptides, which maintain the PG barrier and prevent the release of the pneumolysin toxin (Greene et al. 2015). Unlike the pentaglycine bridge in *S. aureus*, the crossbridge of *S. pneumoniae* mainly consists of two or three alanine residues (Fig. 1A) (Gargis et al. 2009). Streptococci are further subdivided into groups A-V based on the serotyping of specific PG-bound carbohydrate Lancefield antigens. Six of these groups (A, B, C, E, F, and G) are considered clinically relevant (Zorzoli et al. 2019). Cells of group A, represented by *S. pyogenes*, express M protein on their surfaces, which binds numerous plasma proteins involved in complement activation, as well as streptococcal protective antigen (Spa) (Niedermeyer et al. 2014). Streptococci from groups C and G, such as *S. dysgalactiae* subsp. *equisimilis* and *S. equis* subsp. *zooepidemicus*, also possess M proteins highly similar to those of group A (Hashikawa et al. 2004). *S. agalactiae* belongs to group B and expresses surface proteins that play key roles in infection, including C protein, α and Rib proteins of the Alp family, and the LmB lipoproteins (Lindahl et al. 2005). The enterococcal cell wall closely resembles that of staphylococci in composition of the stem peptide, but its crossbridge can contain two alanine residues as in *E. faecalis*, or one D-Asp in *E. faecium* (Fig. 1A). *E. faecalis* PG is crosslinked at about 50% (Yang et al. 2017), with the rate increasing during biofilm formation. The PG in biofilms has more highly crosslinked pentamers, further modified by increased N-deacetylation of NAG and decreased O-acetylation of NAM, which alters the overall charge of the cell and affects interactions with other cells and surfaces. The stem peptide is alanylated by D, D- and L, L-carboxypeptidases, reducing its turnover and enabling modifications (Chang et al. 2018). One of the most prominent surface proteins of *E. faecalis* involved in infection development is the enterococcal surface protein Esp, which has been shown to facilitate biofilm formation (Heikens et al. 2007; Tendolkar et al. 2004). Other surface proteins associated with PG include cytolysin, the enterococcal adhesin Ace, and the endocarditis antigen EfaA (Toledo-Arana et al. 2001).

Regarding Gram-negative bacteria, the most common stem peptide in PG consists of the five-amino acid sequence L-Ala-D-Glu-meso-diaminopimelic acid (meso-DAP)-D-Ala-D-Ala. Adjacent stem peptides are cross-linked directly to each other, creating an abundant 3–4 linkage between position 3 (meso-DAP) and position 4 (D-Ala), or through a less common 3–3 linkage. As noted above, in Gram-positive bacteria, neighbouring peptides may also be connected by an interpeptide bridge (1 − 7 amino acid residues) in addition to direct cross-linking, resulting in 2–4 or 3–4 linkages (Auer and Weibel 2017). Most Gram-negative bacteria produce LPS, which plays a crucial role in the function and structural integrity of the outer lipid membrane and is linked to the pathogenicity of certain bacteria in humans. LPS molecules are negatively charged and consist of three distinct parts: lipid A (the physical anchor between the LPS and the outer lipid membrane), a highly conserved inner and a variable outer polysaccharide core, and a hydrophilic O-antigen. (Campanero-Rhodes et al. 2020)

**Fig. 2:**
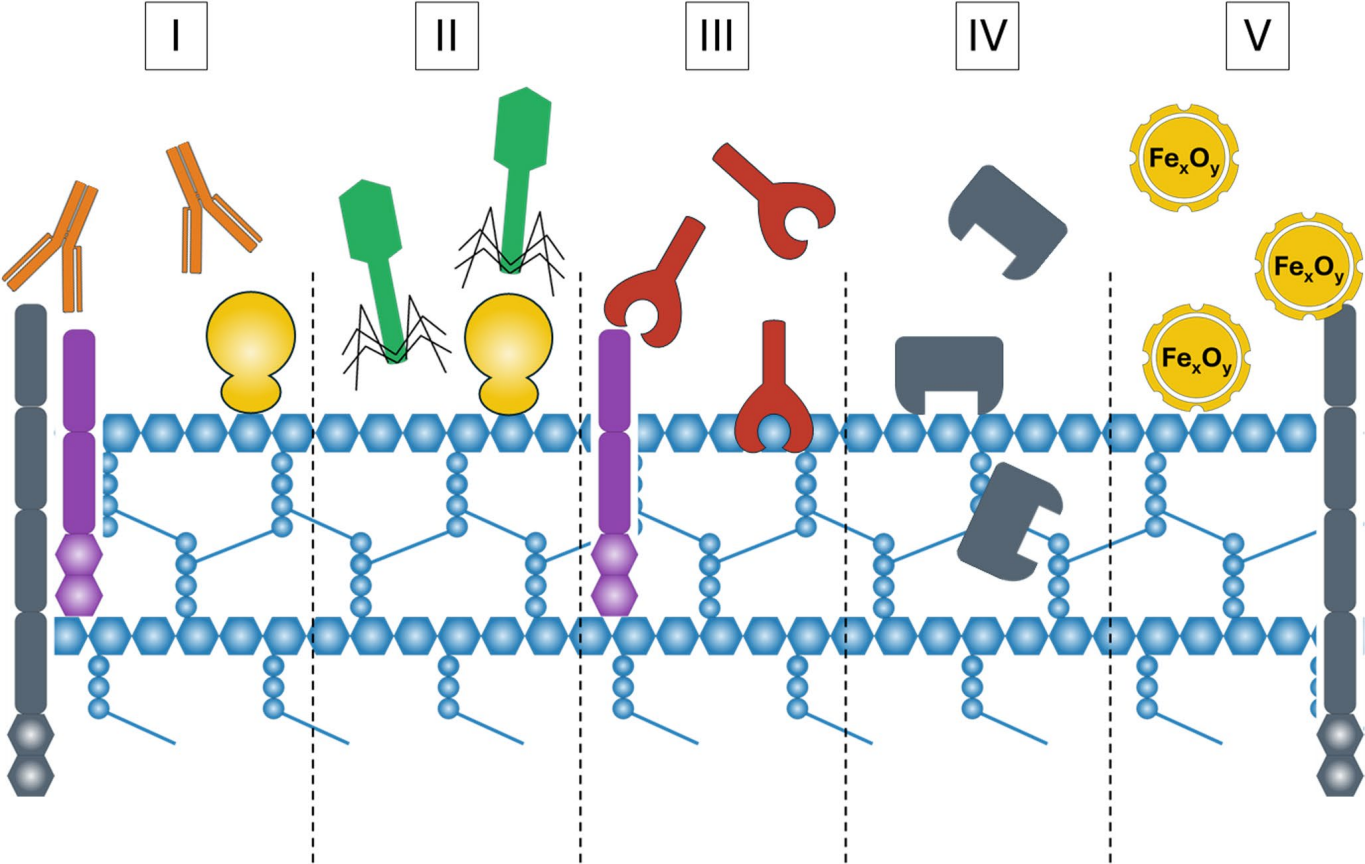
Schematic illustration of agents targeting the bacterial cell wall components and applied for bacterial identification. Broadly used antibodies (I) can target different bacterial cell wall surface molecules. Whole phage particles (II) and phage receptor binding proteins (III) are able to recognize and target pili, flagella, LPS, bacterial surface proteins, peptidoglycan components, (lipo)teichoic acids, or exposed polysaccharides. Such components as N-acetylglucosamine, PG subunits, or secondary cell-wall compounds like choline, polyrhamnose, (lipo)teichoic acids, neutral polysaccharides, and various proteins act as the recognition targets for cell wall-binding domains (IV). Magnetic nanoparticles (V), can bind to different ligands on the bacterial surface depending on functionalized outer coating of them

It should be noted that not all bacteria can be clearly classified as either Gram-negative or Gram-positive. *Corynebacteria* spp., *Mycobacteria* spp., and *Nocardia* spp. have a unique outer membrane called the mycomembrane. This membrane is made up of a thick arabinogalactan–peptidoglycan polymer covalently linked to an outer lipid layer, mainly composed of mycolic acids and arranged in an outer-membrane-like structure. The main components of the mycomembrane include glycolipids, trehalose 6,6’-dimycolate, trehalose monomethylate, and arabinogalactan (Ji et al. 2022). All these bacterial cell wall components that distinguish bacterial cells can serve as markers and may be used for cell detection and identification, as described later in the relevant sections (Fig. 2).

### Immunological techniques for bacteria detection

Among the various techniques used to identify peptidoglycan in bacteria, immunological methods are especially important. The detection of pathogenic bacterial cells through immunological techniques relies on antibody-antigen interactions, where a specific antibody binds to its corresponding antigen on the cell surface (Fig. 2I). These methods allow for the detection of bacteria from the environment, tissue homogenates, and biological fluids such as serum and plasma.

Since the 1970 s, agglutination techniques have been widely used for identifying specific bacterial antigens because they cause antibodies to clump with cells or their parts. The latex agglutination method was employed to identify intrapeptide pentaglycine (Gly₅) bridges in staphylococcal peptidoglycans and trialanine (Ala₃) bridges in micrococcal peptidoglycans, using antisera raised against conjugates of Gly₅ or Ala₃ linked via aminohexanoic acid (Ahx) to albumin (Gly₅–Ahx–albumin and Ala₃–Ahx–albumin, respectively). The most common agglutination tests for bacterial identification rely on lectins (lectin-latex agglutination tests) because of their ability to bind peptidoglycan, teichoic acids, lipopolysaccharides, and capsular materials. Plant lectins, such as Concanavalin A from the jack bean (*Canavalia ensiformis*), which binds to teichoic acids in *S. aureus* and *S. epidermidis*; the lectin from horse gram (*Dolichos biflorus*), which detects group A, B, F, and G streptococci by binding specific carbohydrate residues on their surface; and the lectin from asparagus pea (*Lotus tetragonolobus*), which recognizes fucosyl residues in the cell walls of *Enterococcus faecalis*, have been widely used in bacterial identification (Aitchison et al. 1986a; Archibald and Coapes 1971; Slifkin and Gil 1983). Today, many commercial tests are available to identify Gram-positive bacteria, such as Staphaurex™ Latex Agglutination and Pastorex™ Staph-Plus, which detect clumping factor, Protein A, and capsular polysaccharide in *S. aureus*, or Streptex^®^ Latex Agglutination, which detects Group A, B, C, D, F, and G *Streptococcus*.

The development of immunochemical detection methods and the availability of antibodies enable the detection of bacteria through enzyme-linked immunosorbent assay (ELISA). This technique relies on the color change resulting from an antigen reacting with polyclonal or monoclonal antibodies attached to an appropriate enzyme, which allows for the detection and measurement of specific biomolecules, including bacterial components such as peptidoglycan (Ma et al. 2011; Metwali and Thorne 2010; Yin et al. 2022). The most efficient form is sandwich ELISA, where the primary antibody is immobilized on the well walls of the plate, and an enzyme-conjugated secondary antibody is added after the target antigen, such as bacterial cells, binds to the immobilized primary antibody. The complex formed by the primary antibody, antigen, and secondary antibody is then detected by adding a colorless substrate, which is converted into a colored product in the presence of the enzyme conjugated with the secondary antibody (Zhu et al. 2016).

Antibodies employed in the detection of bacteria include immunoglobulin M antibodies and anti-lipoteichoic acid (anti-LTA) antibodies that recognize Gram-positive bacteria, highly specific anti-(Gly₅) antibodies used for the identification of *S. aureus*, as well as antibodies targeting the cell wall of *Mycobacterium tuberculosis* (Aitchison et al. 1986b; Mauch et al. 1988; Merkel and Scofield 2001; Sandhu et al. 2012). In addition to direct antigen detection, ELISA also enables the quantitative and indirect measurement of peptidoglycans by assessing IgG antibody levels against *S. aureus* peptidoglycan in body fluid samples (Ohsawa et al. 2015; Verbrugh et al. 1981).

Nowadays, ELISA kits along with automated ELISA systems used for rapid detection of foodborne pathogens like *Vibrio parahaemolyticus*,* Salmonella*,* Escherichia coli* O157:H7, *Listeria monocytogenes*,* Campylobacter*,* Bacillus cereus*, and toxins present in foods such as *Clostridium perfringens* toxins, staphylococcal enterotoxins, botulinum toxins, and *E. coli* enterotoxins are currently available (Law et al. 2015; Wang et al. 2015; Zhu et al. 2016). Although immunoassays such as ELISA are very efficient, sensitive, specific, and can be automated, they have a series of limitations, such as being expensive, designed for a limited number of bacterial species, requiring pre-enrichment steps, and necessitating labeling of antibodies or antigens (Franco-Duarte et al. 2019; Law et al. 2015).

## Bacteriophages and their proteins as biorecognition elements

### Whole phage particles

Bacteriophages (or phages) are widespread obligatory viruses that infect and replicate within bacteria. Being highly specific, sensitive, and stable, whole phages can serve as recognition agents for bacterial identification in various detection systems as alternatives to antibodies, nucleic acids, and enzymes (Fig. 2II). Due to the variety of different bacteriophage types, it is theoretically possible to detect nearly every bacterial strain by designing biosensors with phage immobilization or by using free genetically modified phage particles with specific properties, such as displaying fluorescent proteins or enzymes (Paczesny et al. 2020; Hussain et al. 2021).

For example, a non-lytic M13 phage-based electrochemical impedance spectroscopy cytosensor can detect multiple strains of *E. coli* and does not respond to non-*E. coli* bacteria, demonstrating high stability under harsh environmental conditions (Sedki et al. 2020). Additionally, De Plano’s research group used random M13 phage display libraries to select phage clones that specifically bind to the surfaces of *S. aureus*, *Pseudomonas aeruginosa*, and *E. coli* (Plano et al. 2019). Then, phage particles immobilized on magnetic beads were employed for highly sensitive and specific detection of bacteria involved in sepsis using micro-Raman spectroscopy. This detection process takes about 6 h. Furthermore, gold colloid nanoparticles assembled with a P9b phage clone displaying the specific peptide (QRKLAAKLT) were utilized to bind *P. aeruginosa* and detect it through surface-enhanced Raman spectroscopy (Franco et al. 2020). H. Yue and colleagues applied highly specific phage PaP1, depositing phage-conjugated carboxyl graphene onto a glass carbon electrode in an electrochemiluminescent biosensor to detect *P. aeruginosa*, achieving an extremely low detection limit of 56 CFU/mL (Yue et al. 2017). In another example, the specific and selective detection of MRSA, even amidst other major foodborne pathogens such as *E. coli* O157:H7, *L. monocytogenes*, *Salmonella Typhimurium*, *Vibrio parahaemolyticus*, and *Yersinia enterocolitica*, was accomplished using lytic phages immobilized on a magnetoelastic sensor platform (Hiremath et al. 2017). In these two examples, detection was completed within 30 min. Magnetoelastic biosensors are also employed for rapid detection of bacterial cells from the *Salmonella* genus. *S. enterica* and *S. typhimurium* pathogens can be identified in food products at concentrations of 7.86 × 10^5^ CFU/mm² on chicken surface, and 5 × 10² CFU/ml on Romaine lettuce surface, respectively, taking about 2 to 10 min for *Salmonella* detection on food surfaces compared to over 72 h with traditional culture methods (Chen et al. 2017; Mack et al. 2017).

The application of genetically modified phages can be demonstrated with the example of a modified phage T7-ALP expressing alkaline phosphatase. After infecting *E. coli* and collecting them on a 0.22 μm filter membrane, the activity of the overexpressed enzyme causes the development of fluorescence from a specific substrate. This allows for rapid and sensitive detection of bacterial presence through fluorescence imaging in less than 6 h (Wisuthiphaet et al. 2019). Additionally, the overexpression of the fluorescent protein could be performed. Using HK620 and P22 phages, which encode GFP, enables the detection of *E. coli* and *S. enterica* Typhimurium, respectively, with as few as 10 bacterial cells per milliliter by flow cytometry without any concentration or enrichment steps (Vinay et al. 2015).

### Phage proteins

Using phages as sensing units, however, has some drawbacks, such as rapid phage lysis of the target cells, incorrect (random) orientation of virion particles on the surface during immobilization, they can be difficult to purify from host cells and their components, and phage particles are sometimes too large, which may cause to low or reducing signal sensitivity and poor bacterial capture. Additionally, creating modified phages is challenging, and using phages can pose environmental risks to the biosphere if handled improperly (Paczesny et al. 2020; Costa et al. 2023). However, phages are also an excellent source of phage proteins, such as receptor binding proteins (RBPs) and cell-wall binding domains (CBDs) of endolysins. These proteins can recognize different ligands or receptors on the bacterial surface with high specificity, sensitivity, and stability, and can be relatively easy to produce through recombinant overexpression. They can be used as tools for bacterial detection and identification, replacing traditional recognition molecules, or in combination with other techniques (Fig. 2III). For more details about phage proteins used for bacterial detection, we refer readers to the excellent review by Costa et al. (2023). The application of CBDs as recognition molecules for bacterial cell detection will be discussed later in the section on cell wall-binding domains of peptidoglycan hydrolases as biorecognition elements.

Phage RBPs mainly recognize pili, flagella, LPS, or bacterial surface proteins such as porins and transport proteins on Gram-negative bacteria. In Gram-positive bacteria, RBPs target peptidoglycan, (lipo)teichoic acids, or exposed polysaccharides (Filik et al. 2022; Costa et al. 2023). Their use as promising diagnostic tools for pathogen detection has been demonstrated. For example, genetically engineered tailspike proteins from the P22 bacteriophage, immobilized on gold-coated SF-10 glass substrates via Cys-tag, were utilized for real-time detection of *S. enterica* serovar Typhimurium by surface plasmon resonance (SPR), with a detection sensitivity of 10^3^ CFU/mL of bacteria (Singh et al. 2010). In another case, Gp48 RBP from bacteriophage NCTC 12,673, immobilized on gold SPR surfaces, was employed for specific capture of *Campylobacter jejuni*, with a detection limit of approximately 10^2^ CFU/mL on the RBP-derivatized SPR surfaces. *Salmonella* served as a negative control in this study and showed minimal bacterial binding to the Gp48-immobilized surface. GST-Gp48 was also attached to magnetic beads and successfully used to capture and pre-concentrate the host pathogen from suspension (Singh et al. 2011). Brzozowska and colleagues (2016) developed a highly sensitive sensor based on long-period gratings coated with T4 bacteriophage adhesin for detecting Gram-negative bacteria, especially *E. coli* K-12. The recombinant tail fiber protein P069, which can be conjugated with a fluorescent label or immobilized onto magnetic beads, enables detection of *P. aeruginosa* with a detection limit of about 1.7 and 6.7 × 10^2^ CFU/mL using fluorescent and bioluminescent methods, respectively (He et al. 2018a).

RBPs can serve as an initial step and be combined with other molecular techniques for detecting pathogenic bacteria. For instance, RBPs immobilized on magnetic particles can be used for separation and pre-enrichment, providing an alternative to immunomagnetic separation methods. When paired with real-time PCR, this approach has been successfully used for rapid, sensitive, and specific detection of *C. jejuni* in less than 3 h, with a detection limit of 100 CFU/mL (Poshtiban et al. 2013). A common nosocomial pathogen, *Acinetobacter baumannii*, which quickly develops antibiotic resistance, can be detected using bacteriophage ϕAB2 and ϕAB6 tail proteins, such as TF2 and TF6, respectively. These proteins are immobilized on alumina-coated magnetic nanoparticles and can recognize *A. baumannii* clinical isolates M3237 and 54,149 with detection limits around 10^5^ and 10^4^ cells/mL, respectively (Bai et al. 2019). Various bacteriophage tail proteins have been identified as diagnostic tools capable of recognizing other highly pathogenic bacteria, including *Campylobacter* spp., *Yersinia pestis*, *P. aeruginosa*, *L. monocytogenes*, *S. aureus*, *Enterococcus* spp., *Salmonella* spp., *Shigella* spp., *Bacillus anthracis*, and *Klebsiella pneumoniae* (Filik et al. 2022).

## Cell wall-binding domains of peptidoglycan hydrolases as biorecognition elements

Peptidoglycan hydrolases (PGHs) are enzymes that break down peptidoglycan and can be classified into two groups based on their origin: bacterial and viral. Viral-origin enzymes include virion-associated lysins (VALs) and endolysins, which are essential for bacteriophage entry and escape from bacterial cells, respectively. Bacterial-origin enzymes include autolysins, which participate in various physiological processes such as cell wall remodeling during growth, separation and division, biofilm formation, toxin release, sporulation, germination, peptidoglycan recycling, and programmed cell death or autolysis. Additionally, bacterial PGHs include bacteriocins—secreted enzymes used by bacteria to compete and eliminate other bacterial cells (Zhydzetski et al. 2024). In most cases, these enzymes (except VALs) feature a modular structure comprising an enzymatically active domain (EAD), which cleaves specific bonds in peptidoglycan (PG), and a cell wall-binding domain (CBD), which targets, recognizes, and attaches the enzyme to specific ligands on the cell wall near the EAD substrate (Schmelcher et al. 2012). The catalytic domain’s antibacterial activity enables these enzymes to serve as novel antibacterials known as enzybiotics (São-José et al. 2022), while the CBD can be used to recognize and target specific components—often carbohydrate molecules—on the bacterial cell wall.

The presence of a specific target for CBDs within the cell wall is often limited to particular bacterial species or even strains. These species- or strain-specific targets include components of PG itself, such as N-acetylglucosamine, PG subunits, or secondary cell-wall compounds like choline, polyrhamnose, (lipo)teichoic acids, neutral polysaccharides, and proteins (Oliveira et al. 2013; Ganguly et al. 2013; Fischetti 2003), as well as many other bacterial cell wall components essential for bacterial cell viability (Pastagia et al. 2013). Such conserved and vital targets in *Streptococcus pneumoniae* include choline molecules (Doehn et al. 2013); in Group A streptococci, such as *Streptococcus pyogenes*, it is polyrhamnose (Fischetti 2003); and in *B. anthracis*, it is neutral polysaccharides (Pastagia et al. 2013). CBDs bind to these ligands noncovalently with high specificity and affinity, comparable to that of antibodies (Loessner et al. 2002; Yang et al. 2022).

Thus, CBD fused with specific labels such as fluorescent proteins, fluorescent chemical dyes, quantum dots, or other nanoparticles can be used for bacterial species identification, as shown for a series of endolysin CBDs (Fig. 2IV) (Bhagwat et al. 2020). For example, in one of the earliest studies, CBDs from Ply500 and Ply118 endolysins, which lyse *L. monocytogenes*, were fused with green fluorescent protein (GFP). They were able to specifically recognize bacterial cell wall carbohydrates of this species through non-covalent, high-affinity binding with equilibrium association constant values in the nanomolar range (3 × 10^8^–6 × 10^8^) and with 4 × 10^4^ and 8 × 10^4^ binding sites on the cells for CBD118 and CBD500, respectively (Loessner et al. 2002). CTP1L endolysin CBD fused with GFP demonstrated binding to *Clostridium tyrobutyricum* cells when mixed, as observed through fluorescence microscopy (Dunne et al. 2016). Bacteriophage LysP108 protein CBD fused with GFP was used as a signal probe for broad-spectrum fluorimetry of MRSA strains. By employing this bifunctional protein as the signal probe and porcine IgG as the capture agent, MRSA can be detected within a range of 1.0 × 10^3^ to 2.0 × 10^7^ CFU/mL. This approach shows the promising potential of GFP-LysP108-CBD for rapid diagnosis and treatment of MRSA infections and can distinguish this infection from other common food-borne bacteria like *Shigella dysenteriae*, *S. typhimurium*, *L. monocytogenes*, as well as nosocomial bacteria, including *P. aeruginosa* and *E. faecium* (Yang et al. 2022).

The CBD of canonical bacteriocin lysostaphin Lys-Ss fused with GFP was used to identify species-specific CBD ligands in staphylococci. It showed binding only to *S. aureus* strain Newman, causing fluorescence on the cell surface. It had no effect on other Gram-positive bacteria, including *E. faecalis*, *L. monocytogenes*, and *B. subtilis*. It was also shown that variants of *S. aureus* strain Newman lacking the ability to produce polysaccharide capsule, poly-N-acetylglucosamine, lipoproteins, cell wall-anchored proteins, or the glycolipid anchor of lipoteichoic acid bound GFP-CBD similarly to wild-type staphylococci. A *tagO* mutant strain, defective in synthesizing polyribitol wall teichoic acid attached to the cell wall envelope, displayed increased GFP-CBD binding. Conversely, a *femAB* mutation, which reduces both the amount and length of peptidoglycan cross-linking, showed a significant decrease in GFP-CBD binding. This indicates that Lys-Ss CBD directs the enzyme to cross-linked peptidoglycan, which also serves as the substrate for its glycyl-glycine endopeptidase EAD. Though later research using nuclear magnetic resonance spectroscopy showed that, besides binding to pentaglycine, lysostaphin-CBD also binds to the stem peptide and forms a dimer of two contacting CBDs. The pentaglycine crossbridge and the peptide stem are recognized by two independent binding sites located on opposite sides of the SH3b CBD, thus inducing clustering of SH3b domains (Grundling & Schneewind 2006; Gonzales-Delgado et al. 2020). The CBD LysM domain is one of the frequently occurring domains among many prokaryotic, eukaryotic, and viral proteins. It noncovalently binds to N-acetylglucosamine moieties of peptidoglycan and chitin. It has wide specificity, and fusion of the LysM domain with various fluorescent proteins, combined with the use of fluorescence microscopy, has demonstrated its ability to bind to different types of PG in *Bacillus*, *Lactococcus*, *Enterococcus*, *Streptococcus*, and *Lactobacillus* genera (Visweswaran et al. 2014). On the other hand, the specificity of two *Lactobacillus casei* endolysins, Lc-Lys and Lc-Lys-2, was limited to lysing bacterial cells with a D-Asn cross-bridge in their peptidoglycan, such as *Lactococcus casei*, *Lactococcus lactis*, and *E. faecium*. This target specificity extends to the CBD of these two endolysins, which specifically recognize peptidoglycan with a D-Asn cross-bridge. The authors of this study see the potential to identify bacterial species with a D-Asp cross-bridge within a microbial community (Regulski et al. 2013).

The binding of RBPs and CBDs to PG and other cell wall components is fast, robust, and strong. Compared to other sensing molecules like nucleic acids, enzymes, antibodies, and aptamers, these proteins and their conjugates have high thermal and pH stability, resist proteases and nucleases, are easy to genetically and chemically modify, have low production costs, and offer greater flexibility and nonlytic properties compared to intact phages (He et al. 2018a; Costa et al. 2023; Singh et al. 2010).

Some studies showed the potential and imminent practical application of CBD in lateral flow assays (LFA) for cost-effective and efficient bacterial detection. This was demonstrated with pathogenic bacteria Bacillus cereus using LFA strips that had a detection limit of 1 × 10^4 CFU/mL and an overall assay time of about 20 min, which outperformed traditional antibody-based methods (Kong et al. 2017). LFA can also utilize other tracer molecules like antibodies, labeled phages, and engineered phage proteins (Liu et al. 2023). Additionally, RBPs and CBDs as detecting agents can be conjugated to magnetic nanoparticles and integrated with biosensor techniques, as described in the next section. These methods now serve as prototypes for detecting *Bacillus cereus* and *Acinetobacter baumannii* (Park et al. 2018; Yu et al. 2025).

### Magnetic nanoparticles bioconjugates

The use of magnetic nanoparticles (MNPs) in medicine and diagnostics has been extensively researched (Fig. 2V). MNPs allow for quick, accurate, and highly specific detection of pathogens (Niemirowicz et al. 2012) and cancer cells (Alirezaie Alavijeh et al. 2019). They consist of an iron oxide core and an external coating. The core is typically made of magnetite (Fe_3_O_4_), hematite (α-Fe_2_O_3_), or maghemite (γ-Fe_2_O_3_) (Farinha et al. 2021). MNPs exhibit superparamagnetic properties, meaning they become magnetized only when exposed to an external magnetic field. This trait makes MNPs ideal for biological applications because they are easy to manipulate and do not spontaneously aggregate (Rarokar et al. 2024). The coating material varies depending on the final application of the MNPs, as it provides a surface for attaching additional ligands. Inorganic materials such as gold, graphene, or silica enable particle functionalization, improve stability, and prevent oxidation. Polymeric coatings (like polyethene, alginate, or dextran) enhance biocompatibility, allowing these particles to be injected into the bloodstream later (Farinha et al. 2021). Functionalizing the outer coating of MNPs is crucial for targeting specific objects. Various approaches have been developed based on the type of object being targeted and the intended use. MNP surfaces can be functionalized by attaching antibodies, aptamers, peptides, or other ligands. Bacteria-MNP complexes can then be separated using a magnetic field and analyzed via PCR, Raman spectroscopy, or fluorescent microscopy (Pan et al. 2012).

Abafogi et al. (2022) studied MNPs capable of binding to bacteria that cause sepsis. Coating the MNPs with polydopamine (PDA) prevented particle aggregation in solution and created anchors for vancomycin molecules. Vancomycin binds to the D-Ala-D-Ala terminus of the stem peptide in the peptidoglycan of Gram-positive bacteria. Van-PDA-MNPs were used to preconcentrate bacteria in blood samples, which were then identified using PCR and qPCR. The authors suggest that combining van-PDA-MNPs with PDA-MNPs conjugated with polymyxin B (which recognizes Gram-negative bacteria) could enable more comprehensive diagnostics. Isolating the pathogen with PDA-MNPs may be a first step in removing and heat-inactivating *S. aureus* cells from various samples (Li et al. 2022). Another diagnostic method was developed by Lim et al. (2019), where the surface of MNPs was modified with peptidoglycan binding protein (PBP) for specific capture of *S. aureus* in blood samples. Additionally, the PBP binds universally to peptidoglycans of various Gram-positive bacteria, such as *Bacillus*. PBP-MNPs are more practical than probes based on antibodies. Huang et al. (2010) used amine-functionalized MNPs (AF-MNPs) to capture bacterial cells from water, beverages, and urine samples. The presence of amines on the surface of MNPs creates a positive charge, which promotes strong interactions with negatively charged bacterial surfaces. The removal of bacteria from water, food, and urine samples was quick and effective, with efficiencies ranging from 88.5% to 99.1%. Compared to MNPs conjugated with antibodies or peptides, AF-MNPs are cheaper and less fragile.

Some functionalized MNPs exhibit additional properties, such as bactericidal activity. Ceragenins are synthetic compounds that mimic antimicrobial peptides and can be somewhat toxic to host cells. The C-13-MNPs complex maintained its bactericidal activity against *P. aeruginosa*, while their biocompatibility increased, making the conjugates safer for use in humans (Niemirowicz et al. 2015). Friedrich et al. developed a system that aggregates Gram-positive bacteria by recognizing LTA. Peptides attached to the MNPs were derived from the innate immune receptor GP-340 (Friedrich et al. 2022).

Besides the approaches and proteins described above as biorecognition agents, techniques such as using fluorescent probes to identify bacteria—which recognize various pathogen targets—and focusing on metabolic labeling of pathogenic bacteria surface components are also employed. These include identifying cell wall components, binding to pathogenic endogenous enzymes, and nonspecific detection. (Ji et al. 2022) Other methods involve glycopeptide antibiotics like vancomycin and polymyxin, which have high affinity for D-Ala-D-Ala dipeptides on bacterial cell walls; lectin proteins that specifically interact with carbohydrates (mono- and oligosaccharides) on bacterial surfaces; antimicrobial peptides and various proteins (such as human serum albumin, lactoferrin, lysozyme) that possess specific receptors on bacterial surfaces and are used in array-based biosensors. Additionally, conjugated polymers with different structural designs can also be utilized for bacterial identification (Yu and Xianyu 2021).

## Conclusion and perspectives

Despite the variety and high efficiency of existing methods and kits for detecting pathogenic bacteria, each with its own advantages and drawbacks, there remains a need for faster, cheaper, and more reliable microbial identification techniques. This is especially critical in the era of antibiotic-resistant bacteria, where quick and preliminary pathogen detection can save time and lives. Potential biorecognition agents could include phage-derived proteins, such as RBDs or CBDs of peptidoglycan hydrolases, also known as endolysins. These agents could be fused with reporter probes and would have high affinity and specificity for bacterial cell wall surface ligands, along with high sensitivity and stability under various conditions. The abundance of viruses and bacteria as sources of RBPs and CBDs offers an opportunity to create and expand a comprehensive database of these proteins as biorecognition agents, enabling the development of detection kits for any pathogen if needed. Moreover, gene and protein engineering techniques facilitate the creation of more specific, sensitive, and stable molecules with improved binding properties as targeted recognition tools. Although current CBD- or RBD-based detection studies are still in the research and development phase and primarily consist of laboratory prototypes, they demonstrate significant potential as biorecognition agents on natural and real-world samples. Some of these advancements are nearing practical application, and integrating them with other biosensing platforms or lateral flow assays is likely to become more common in clinical diagnostics, such as detecting *A. baumannii*, *B. cereus*, *S. enterica*, and *S. aureus*. Additionally, these methods can be further refined to detect other pathogens.
